# Phenotypic and Biochemical Responses and Drought Resistance of *Allium polyrhizum* to Drought Stress

**DOI:** 10.3390/plants15172646

**Published:** 2026-08-29

**Authors:** Shaochong Wei, Huinan Wang, Shaopeng Chen, Liang Mao, Xiaojun Yu

**Affiliations:** Key Laboratory of Grassland Ecosystem, Ministry of Education/Sino, U.S. Center for Grazing Land, College of Grassland Science, Gansu Agricultural University, Lanzhou 730070, China; weisc@st.gsau.edu.cn (S.W.);

**Keywords:** drought stress, *Allium polyrhizum*, phenotypic characteristics, biochemical indicators, membership function

## Abstract

*Allium polyrhizum* is an important native species in arid and semi-arid regions. This study investigated the responses of *A. polyrhizum* to drought stress and screened drought-resistant germplasms to provide a basis for the selection of high-quality germplasm resources for degraded grassland restoration. Five *A. polyrhizum* germplasms were collected from Longquansi Town (LQS), Hongcheng Town (HC), Zhongchuan Town (ZC), and Zhonghe Town (ZH-P and ZH-W) in Lanzhou, Gansu Province. The phenotypic and biochemical indicators were measured under the control treatment (CK) and PEG-6000-simulated drought stress treatments at −0.2, −0.6, −1.0, −1.4, and −1.8 MPa. The results showed that, with increasing drought stress intensity, the root length, shoot height, aboveground biomass, and belowground biomass of the five *A. polyrhizum* germplasms generally decreased, whereas the contents of soluble protein, soluble sugar, free proline, and malondialdehyde generally increased. The activities of superoxide dismutase, peroxidase, and catalase generally increased initially and then decreased, although the magnitude of these responses varied among germplasms. Two-way ANOVA showed that drought stress and germplasm had highly significant effects on all 11 measured indicators, whereas their interaction had highly significant effects on most indicators. The drought resistance coefficients of the different indicators were correlated to varying degrees. Comprehensive evaluation showed that the drought resistance of the five germplasms was ranked as ZH-W > HC > ZH-P > ZC > LQS.

## 1. Introduction

Global warming has intensified extreme environmental conditions such as drought, placing increasing pressure on ecosystems across different regions [1,2,3]. Drought stress can affect multiple aspects of plant function, including cell membrane integrity, osmotic adjustment systems, and photosynthetic capacity [3,4,5]. Xerophytes can adapt to drought conditions through a series of morphological, physiological, and biochemical adjustments, thereby maintaining normal growth and survival [6,7,8,9]. *Allium polyrhizum*, a perennial herbaceous plant, is a representative forage species. As a typical xerophyte, it is mainly distributed in arid and semi-arid grasslands of Eastern Siberia, Central Asia, Mongolia, and several provinces in China, including Henan, Shanxi, Ningxia, Gansu, Qinghai, and Xinjiang [10]. Following the degradation of typical steppes dominated by *Stipa krylovii*, *A. polyrhizum* gradually becomes the dominant species [11]. As a dominant species in xerophytic desert steppes, *A. polyrhizum* has evolved a sophisticated suite of drought escape mechanisms. For example, during drought periods that normally fall within its growing season, it can remain dormant and delay germination, thereby reducing the adverse effects of drought [12]. In drought environments, the root system of plants can perceive changes in the external environment and coordinate with the aboveground parts to maintain normal growth and development under adverse conditions [13].

At present, research on *A. polyrhizum* mainly focuses on its total flavonoid content [14], the effects of nitrogen and phosphorus addition on biomass [15], and interspecific competition [16], whereas relatively little attention has been paid to its stress tolerance. Previous studies have found that the roots and leaves of *A. polyrhizum* possess well-developed water-storage tissues, classifying it as a succulent, drought-tolerant plant [17]. A study on the ultrasonic extraction and antioxidant activity of total flavonoids from *A. polyrhizum* showed that antioxidant activity in the plant reflects its adaptability to adverse environments [18]. However, studies on the drought resistance of different *A. polyrhizum* germplasms remain scarce.

Therefore, five *A. polyrhizum* germplasms collected from Lanzhou were used as experimental materials. PEG-6000 solutions with different osmotic potentials were applied to simulate drought stress conditions. Drought resistance among the five germplasms was compared based on changes in phenotypic and biochemical characteristics under different levels of drought stress, providing a basis for selecting high-quality germplasm resources for grassland improvement in harsh habitats of arid and semi-arid regions.

## 2. Results

### 2.1. Effects of Drought Stress on the Phenotypic Characteristics of A. polyrhizum Germplasms

#### 2.1.1. Effects of Drought Stress on Shoot Height and Root Length of *A. polyrhizum* Germplasms

As shown in Table 1, both shoot height and root length of the five *A. polyrhizum* germplasms generally decreased as drought stress intensified. The shoot heights of LQS, HC, ZC, ZH-P, and ZH-W under −1.8 MPa were significantly lower than those under the CK treatment (*p* < 0.05), decreasing by 43.1%, 26.1%, 62.9%, 50.4%, and 58.6%, respectively, compared with CK. Under the same drought stress level, ZH-P generally exhibited the greatest shoot height among the five germplasms, with ZH-P and ZH-W showing the same shoot height under −0.2 MPa. Under −1.8 MPa, the root lengths of LQS, HC, ZC, ZH-P, and ZH-W decreased by 54.3%, 32.4%, 20.5%, 49.1%, and 53.4%, respectively, compared with CK. The reductions were significant for LQS, ZH-P, and ZH-W (*p* < 0.05), whereas no significant differences were detected among drought stress treatments for HC and ZC (*p* > 0.05). Under the same drought stress level, HC consistently exhibited the greatest root length among the five germplasms across all treatments.

#### 2.1.2. Effects of Drought Stress on Aboveground and Belowground Biomass of *A. polyrhizum* Germplasms

As shown in Table 2, both aboveground and belowground biomass of the five *A. polyrhizum* germplasms decreased as drought stress intensified. The aboveground biomass of LQS, HC, ZC, ZH-P, and ZH-W under −1.8 MPa was significantly lower than that under the CK treatment (*p* < 0.05), decreasing by 72.4%, 75.4%, 89.4%, 69.9%, and 70.2%, respectively, compared with CK. Under the same drought stress level, ZC consistently exhibited the highest aboveground biomass among the five germplasms from CK to −1.4 MPa. The belowground biomass of LQS, HC, ZC, ZH-P, and ZH-W under −1.8 MPa was also significantly lower than that under the CK treatment (*p* < 0.05), decreasing by 68.5%, 65.2%, 68.9%, 70.7%, and 60.1%, respectively, compared with CK. At the same drought stress, HC exhibited the highest belowground biomass among the five germplasms under all treatments except −1.4 MPa.

### 2.2. Effects of Drought Stress on the Biochemical Characteristics of A. polyrhizum Germplasms

#### 2.2.1. Effects of Drought Stress on Antioxidant Enzyme Activities of *A. polyrhizum* Germplasms

**Figure 1:**
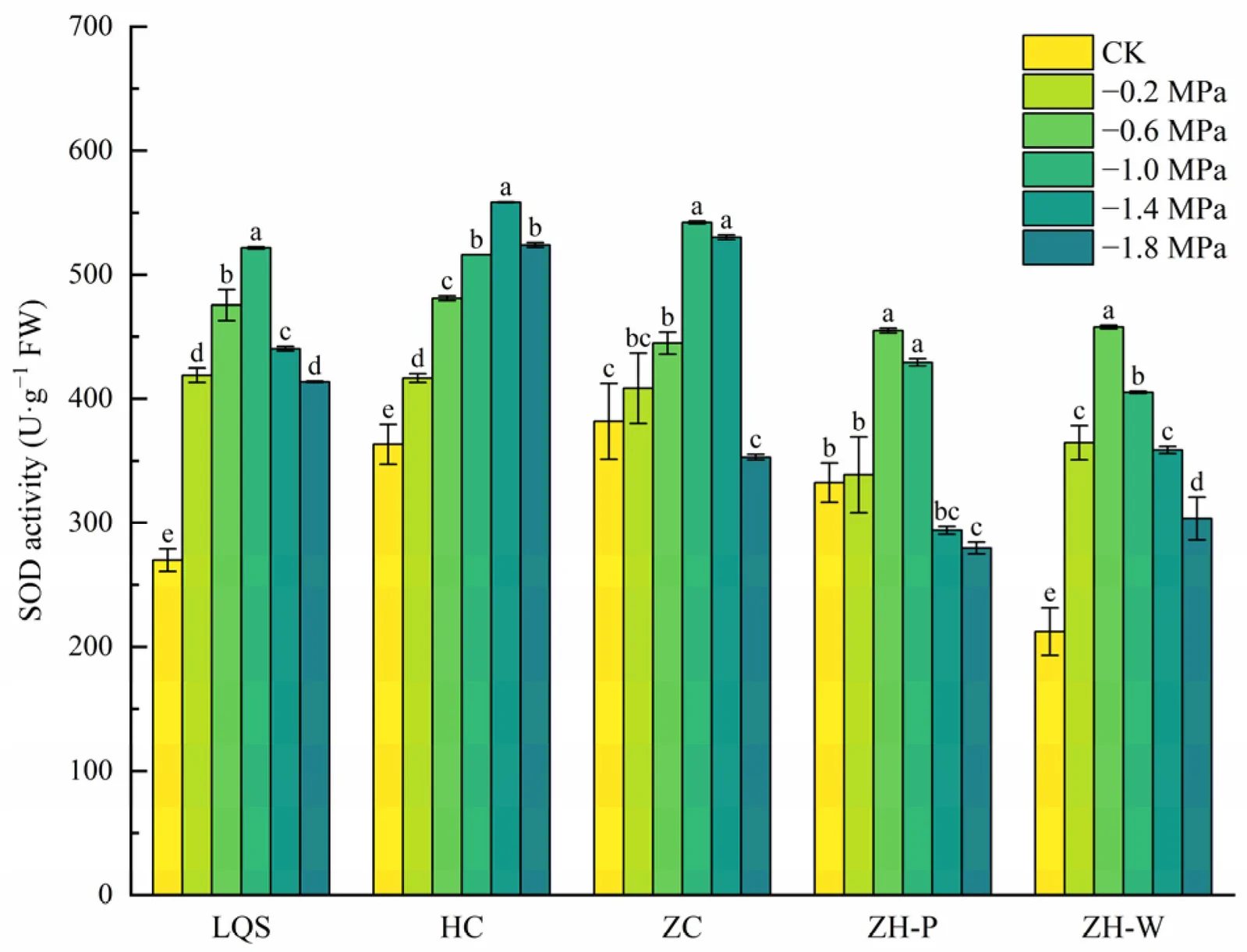
Effects of drought stress on superoxide dismutase activity in *A. polyrhizum* germplasms. LQS, Longquansi; HC, Hongcheng; ZC, Zhongchuan; ZH-P and ZH-W, Zhonghe, represent the collection sites of the *A. polyrhizum* germplasms and were used to designate the different germplasms. Different lowercase letters indicate significant differences among treatments within the same germplasm (*p* < 0.05), whereas the same letter indicates no significant difference.

Different stress treatments significantly affected the superoxide dismutase (SOD) activity of the five *A. polyrhizum* germplasms (*p* < 0.05) (Figure 1). As drought stress increased, SOD activity in all five germplasms generally showed an initial increase followed by a decrease, although the stress treatment at which peak activity occurred differed among germplasms. SOD activity peaked at −1.0 MPa in LQS and ZC, at −0.6 MPa in ZH-P and ZH-W, and at −1.4 MPa in HC. The changes in SOD activity also differed among the five germplasms as drought stress increased. LQS and ZH-W showed the greatest increases from CK to −0.2 MPa, with SOD activity increasing by 55.2% and 71.7%, respectively. HC and ZH-P exhibited the greatest increases from −0.2 to −0.6 MPa, with increases of 15.5% and 34.3%, respectively. ZC showed the greatest increase from −0.6 to −1.0 MPa, with an increase of 21.9%, whereas its greatest decrease occurred from −1.4 to −1.8 MPa, with a reduction of 33.4%.

**Figure 2:**
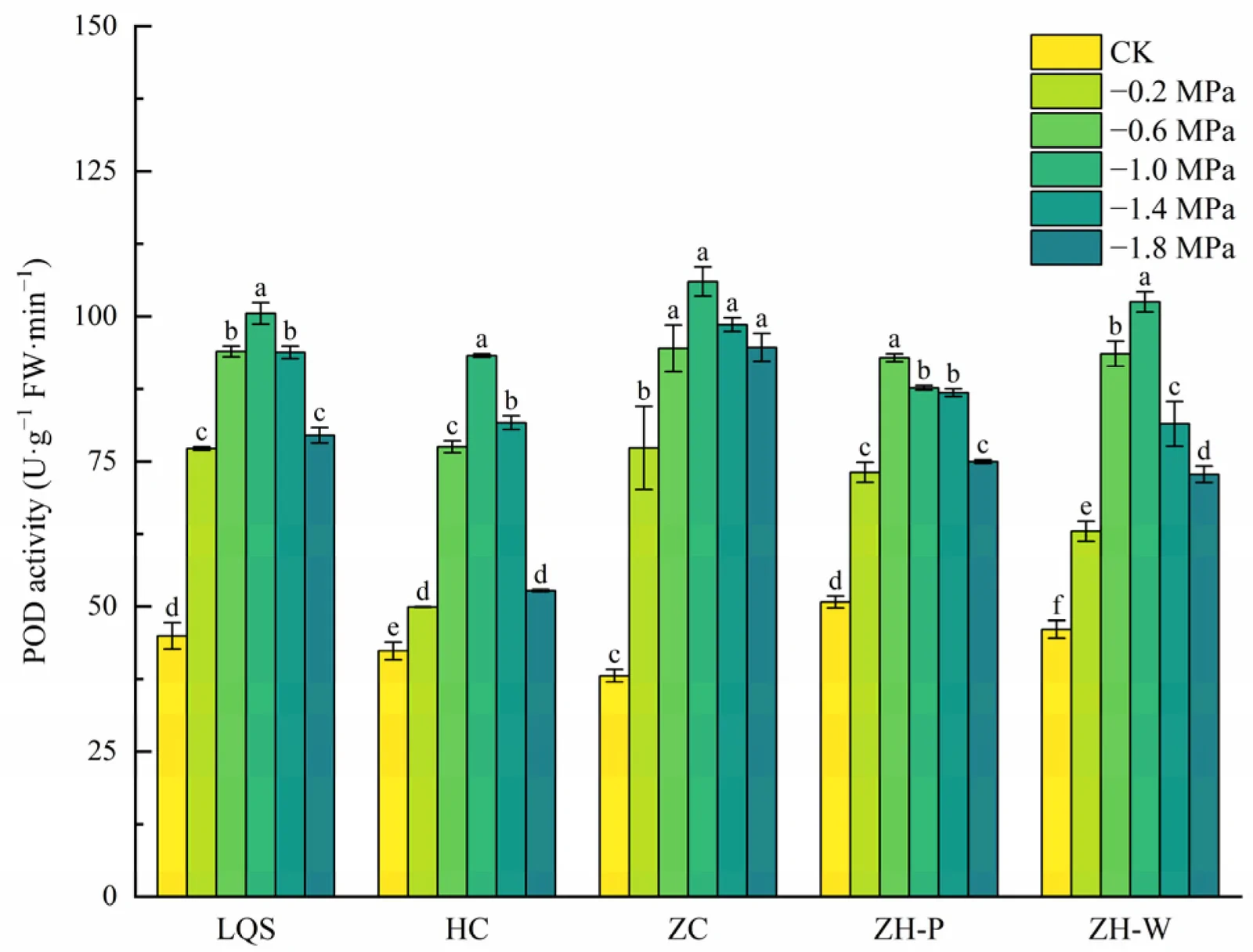
Effects of drought stress on peroxidase activity in *A. polyrhizum* germplasms. LQS, Longquansi; HC, Hongcheng; ZC, Zhongchuan; ZH-P and ZH-W, Zhonghe, represent the collection sites of the *A. polyrhizum* germplasms and were used to designate the different germplasms. Different lowercase letters indicate significant differences among treatments within the same germplasm (*p* < 0.05), whereas the same letter indicates no significant difference.

Different stress treatments significantly affected peroxidase (POD) activity in the five *A. polyrhizum* germplasms (*p* < 0.05) (Figure 2). As drought stress increased, POD activity in all five germplasms generally showed an initial increase followed by a decrease. POD activity peaked at −1.0 MPa in LQS, HC, ZC, and ZH-W, whereas it peaked at −0.6 MPa in ZH-P. LQS, ZC, and ZH-P showed their greatest increases from CK to −0.2 MPa, with POD activity increasing by 71.9%, 103.1%, and 44.0%, respectively; their greatest decreases occurred from −1.4 to −1.8 MPa, −1.0 to −1.4 MPa, and −1.4 to −1.8 MPa, respectively, with reductions of 15.2%, 7.0%, and 13.7%. HC and ZH-W exhibited their greatest increases from −0.2 to −0.6 MPa, with increases of 55.3% and 48.5%, respectively; their greatest decreases occurred from −1.4 to −1.8 MPa and −1.0 to −1.4 MPa, respectively.

**Figure 3:**
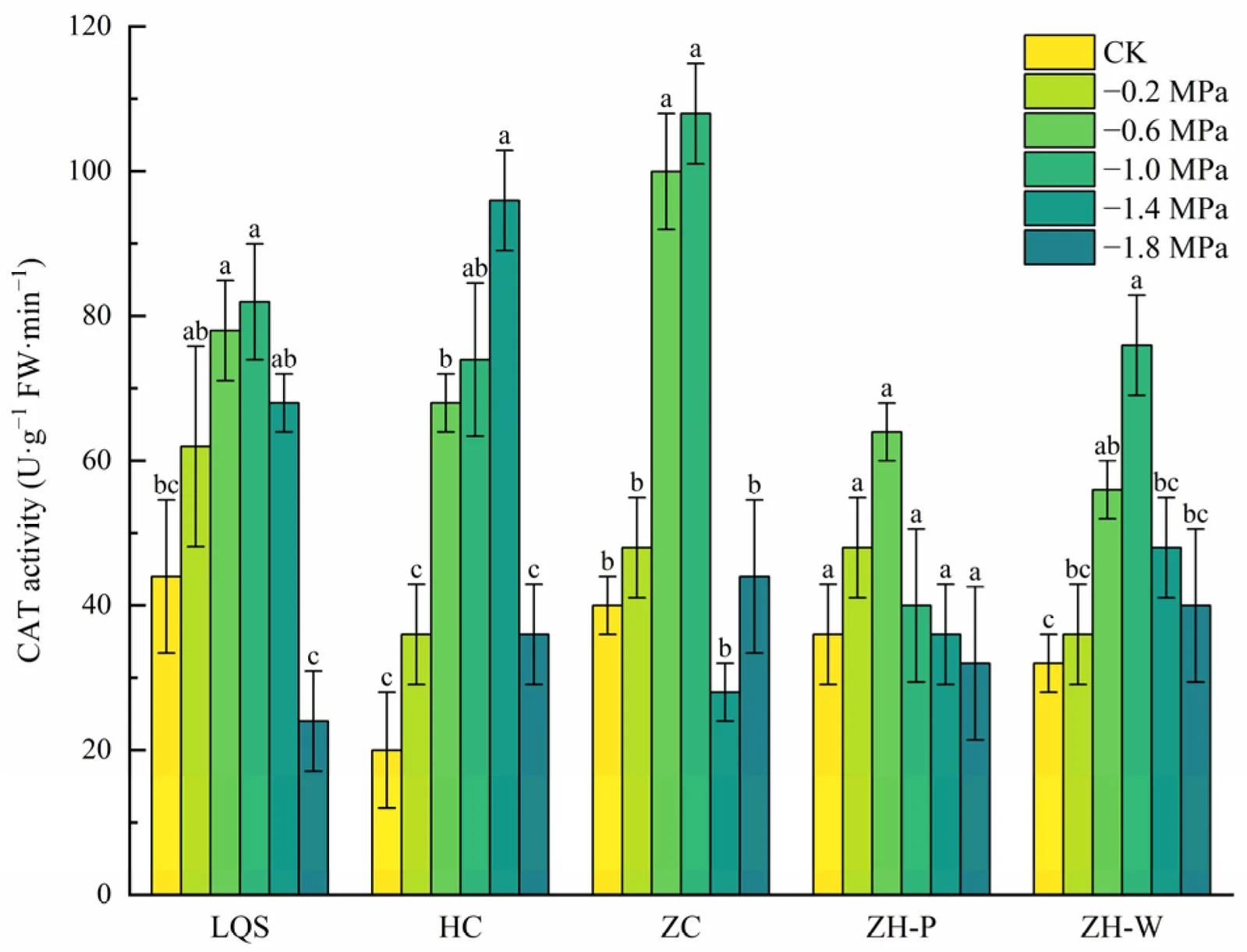
Effects of drought stress on catalase activity in *A. polyrhizum* germplasms. LQS, Longquansi; HC, Hongcheng; ZC, Zhongchuan; ZH-P and ZH-W, Zhonghe, represent the collection sites of the *A. polyrhizum* germplasms and were used to designate the different germplasms. Different lowercase letters indicate significant differences among treatments within the same germplasm (*p* < 0.05), whereas the same letter indicates no significant difference.

Different stress treatments significantly affected catalase (CAT) activity in LQS, HC, ZC, and ZH-W *A. polyrhizum* germplasms (*p* < 0.05) (Figure 3). As drought stress increased, CAT activity in all five germplasms generally showed an initial increase followed by a decrease. CAT activity peaked at −1.0 MPa in LQS, ZC, and ZH-W, at −0.6 MPa in ZH-P, and at −1.4 MPa in HC. LQS showed its greatest increase from CK to −0.2 MPa and its greatest decrease from −1.4 to −1.8 MPa, with an increase of 40.9% and a reduction of 64.7%, respectively. HC, ZC, ZH-P, and ZH-W all showed their greatest increases from −0.2 to −0.6 MPa, with increases of 88.9%, 108.3%, 33.3%, and 55.5%, respectively; their greatest decreases occurred from −1.4 to −1.8 MPa, −1.0 to −1.4 MPa, −0.6 to −1.0 MPa, and −1.0 to −1.4 MPa, respectively, with reductions of 62.5%, 74.1%, 37.5%, and 36.8%.

#### 2.2.2. Effects of Drought Stress on Osmotic Adjustment Substances of *A. polyrhizum* Germplasms

**Figure 4:**
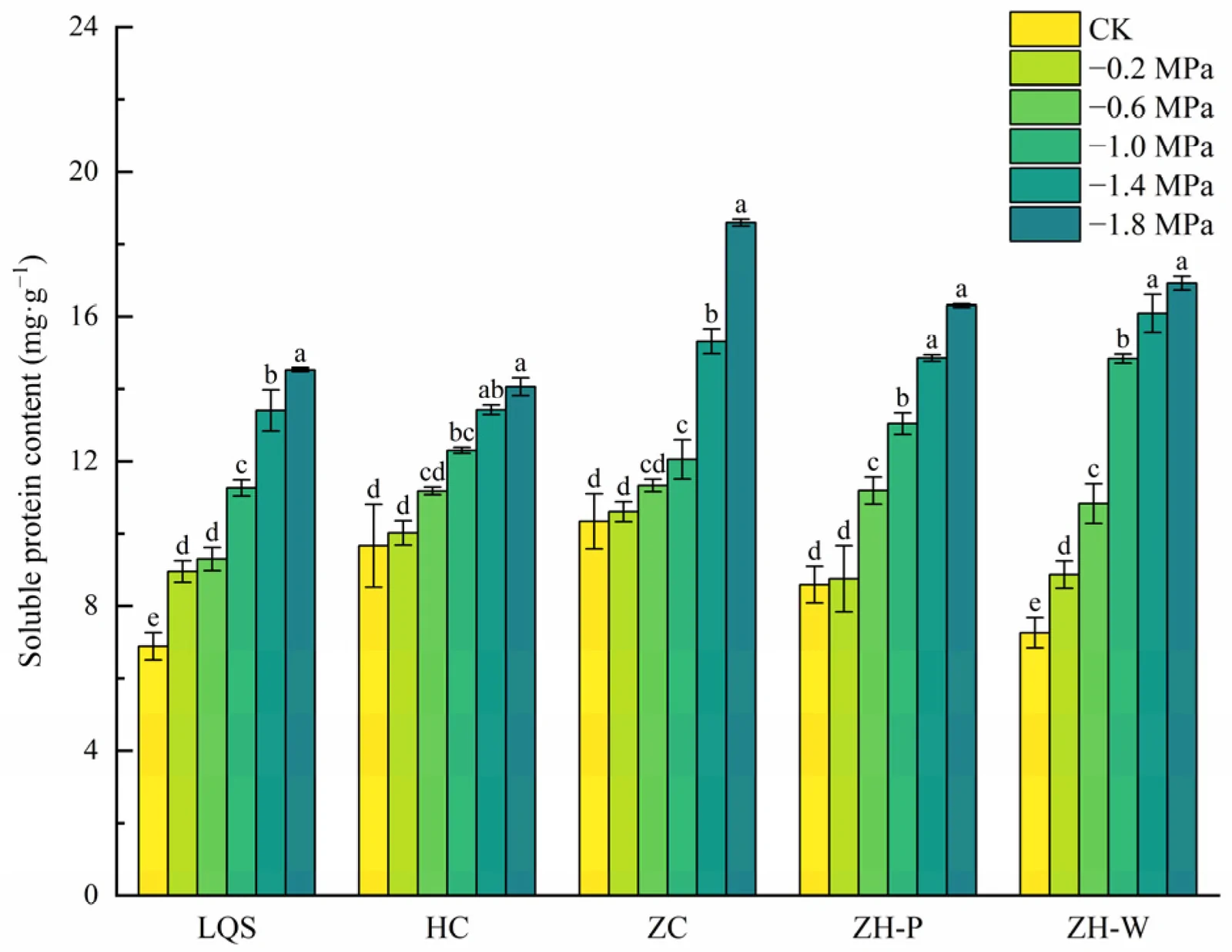
Effects of drought stress on soluble protein content in *A. polyrhizum* germplasms. LQS, Longquansi; HC, Hongcheng; ZC, Zhongchuan; ZH-P and ZH-W, Zhonghe, represent the collection sites of the *A. polyrhizum* germplasms and were used to designate the different germplasms. Different lowercase letters indicate significant differences among treatments within the same germplasm (*p* < 0.05), whereas the same letter indicates no significant difference.

Different stress treatments significantly affected soluble protein content in the five *A. polyrhizum* germplasms (*p* < 0.05) (Figure 4). As drought stress increased, soluble protein content in all five germplasms generally showed an increasing trend and peaked at −1.8 MPa. LQS and ZC showed their greatest increases from CK to −0.2 MPa and from −1.0 to −1.4 MPa, with increases of 30.0% and 27.1%, respectively. HC and ZH-P exhibited their greatest increases from −0.2 to −0.6 MPa, with increases of 11.6% and 27.9%, respectively. ZH-W showed its greatest increase from −0.6 to −1.0 MPa, with an increase of 37.0%.

**Figure 5:**
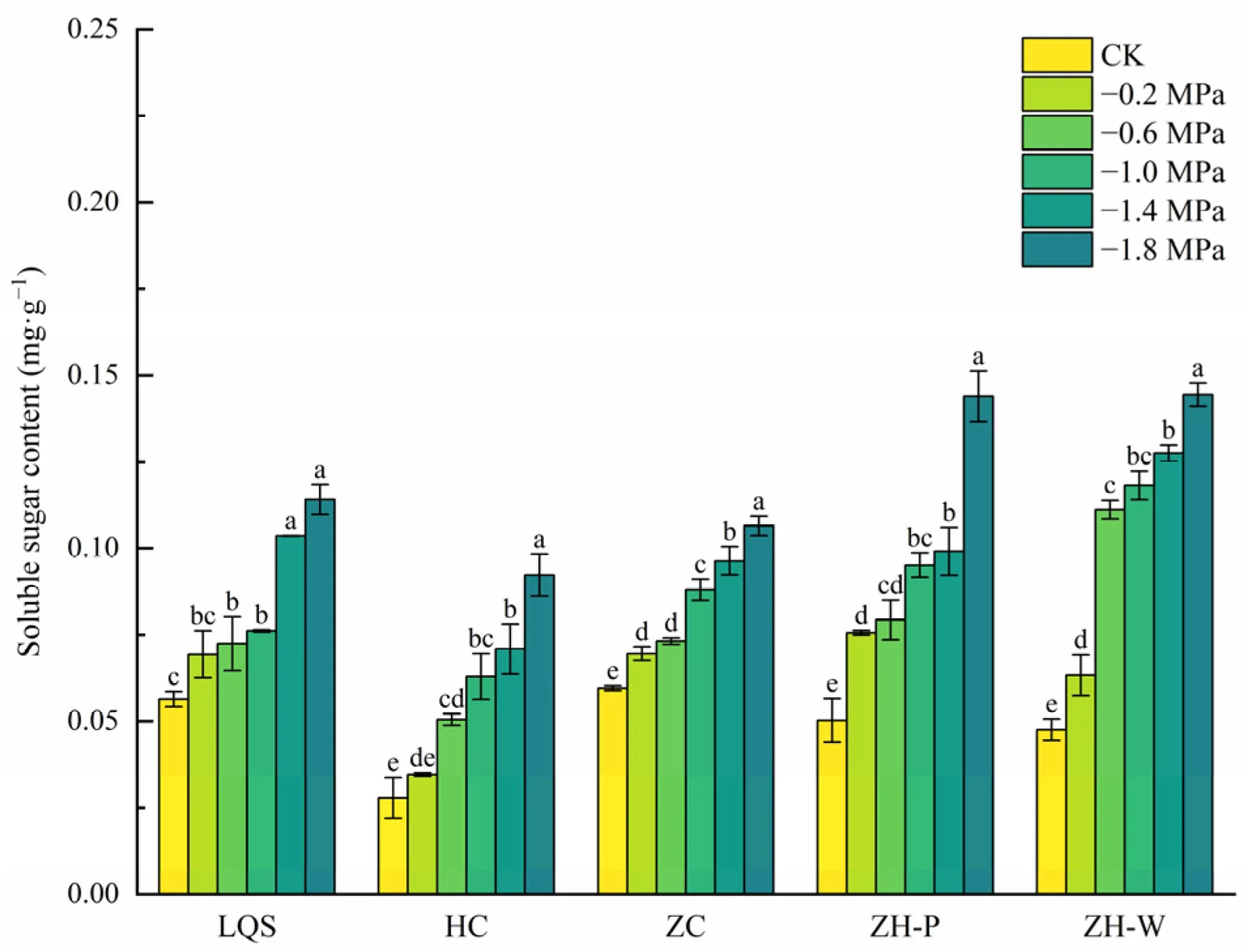
Effects of drought stress on soluble sugar content in *A. polyrhizum* germplasms. LQS, Longquansi; HC, Hongcheng; ZC, Zhongchuan; ZH-P and ZH-W, Zhonghe, represent the collection sites of the *A. polyrhizum* germplasms and were used to designate the different germplasms. Different lowercase letters indicate significant differences among treatments within the same germplasm (*p* < 0.05), whereas the same letter indicates no significant difference.

Different stress treatments significantly affected soluble sugar content in the five *A. polyrhizum* germplasms (*p* < 0.05) (Figure 5). As drought stress increased, soluble sugar content in all five germplasms generally showed an increasing trend and peaked at −1.8 MPa. LQS showed its greatest increase from −1.0 to −1.4 MPa, with an increase of 36.1%. HC showed its greatest increase from −0.2 to −0.6 MPa, with an increase of 45.9%. ZC showed its greatest increase from −0.6 to −1.0 MPa, with an increase of 20.3%. ZH-P showed its greatest increase from CK to −0.2 MPa, with an increase of 50.4%, whereas ZH-W showed its greatest increase from −0.2 to −0.6 MPa, with an increase of 75.6%.

**Figure 6:**
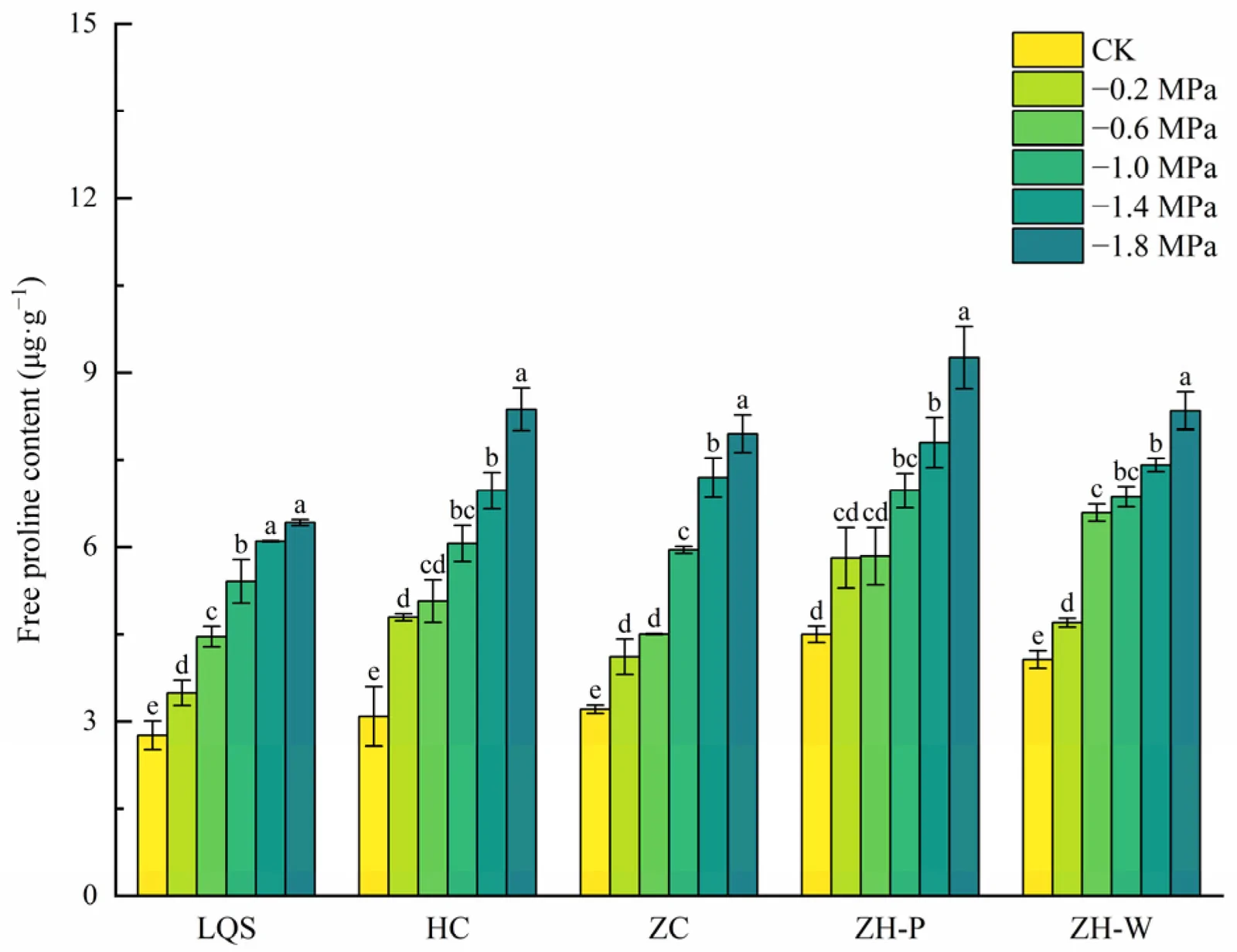
Effects of drought stress on free proline content in *A. polyrhizum* germplasms. LQS, Longquansi; HC, Hongcheng; ZC, Zhongchuan; ZH-P and ZH-W, Zhonghe, represent the collection sites of the *A. polyrhizum* germplasms and were used to designate the different germplasms. Different lowercase letters indicate significant differences among treatments within the same germplasm (*p* < 0.05), whereas the same letter indicates no significant difference.

Different stress treatments significantly affected free proline content in the five *A. polyrhizum* germplasms (*p* < 0.05) (Figure 6). As drought stress increased, free proline content in all five germplasms generally showed an increasing trend and peaked at −1.8 MPa. Comparison of the increases in proline content among the five germplasms showed that, from CK to −0.2 MPa, HC exhibited the greatest increase; from −0.2 to −0.6 MPa, ZH-W showed the greatest increase, with an increase of 40.3%; from −0.6 to −1.0 MPa and from −1.0 to −1.4 MPa, ZC exhibited the greatest increases; from −1.4 to −1.8 MPa, LQS showed the smallest increase, whereas HC showed the greatest increase, with increases of 5.3% and 20.1%, respectively.

#### 2.2.3. Effects of Drought Stress on Membrane Lipid Peroxidation in *A. polyrhizum* Germplasms

**Figure 7:**
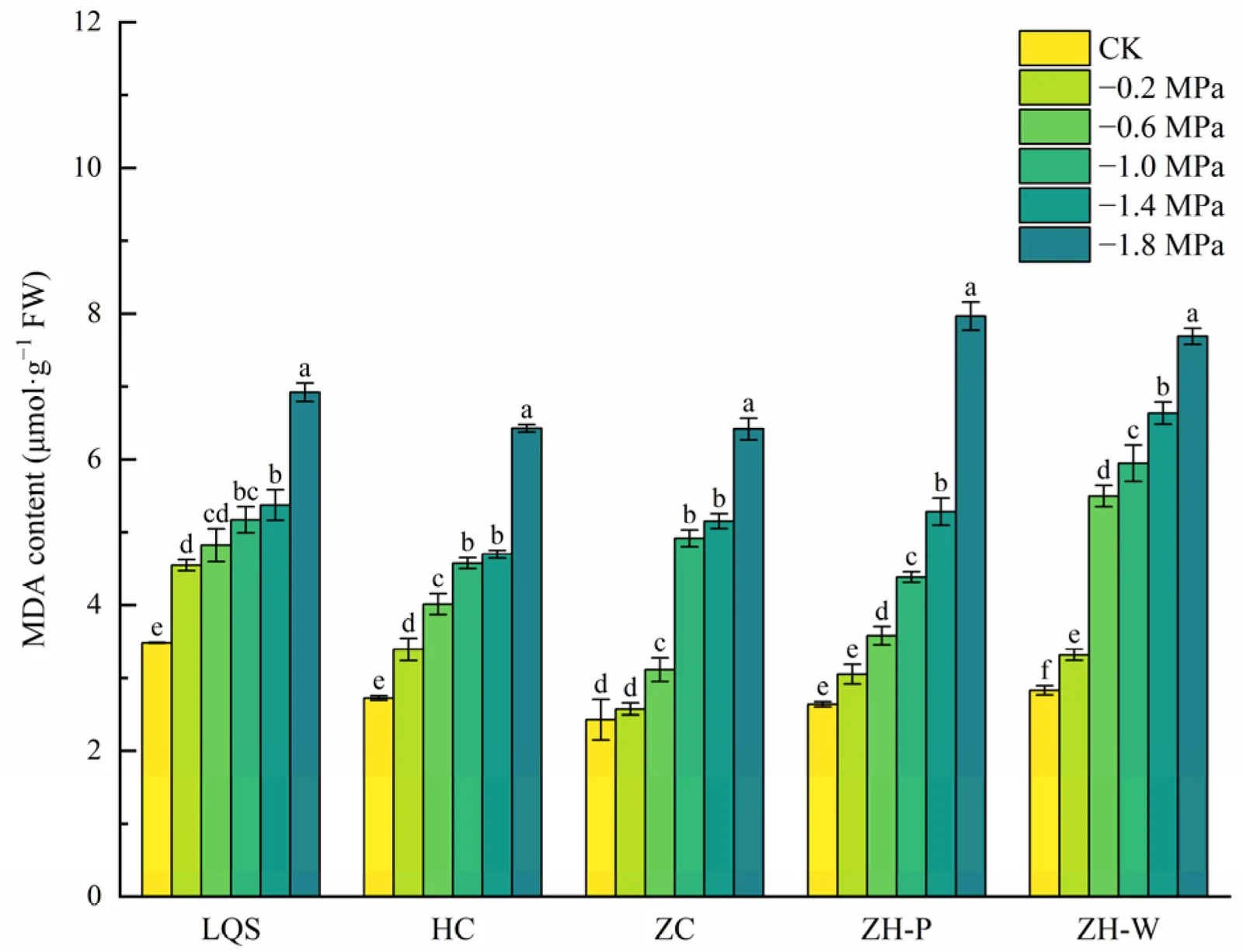
Effects of drought stress on malondialdehyde content in *A. polyrhizum* germplasms. LQS, Longquansi; HC, Hongcheng; ZC, Zhongchuan; ZH-P and ZH-W, Zhonghe, represent the collection sites of the *A. polyrhizum* germplasms and were used to designate the different germplasms. Different lowercase letters indicate significant differences among treatments within the same germplasm (*p* < 0.05), whereas the same letter indicates no significant difference.

Different stress treatments significantly affected malondialdehyde (MDA) content in the five *A. polyrhizum* germplasms (*p* < 0.05) (Figure 7). As drought stress increased, MDA content in all five germplasms generally showed an increasing trend and peaked at −1.8 MPa. LQS showed its greatest increase from CK to −0.2 MPa, with an increase of 30.7%. HC and ZH-P showed their greatest increases from −1.4 to −1.8 MPa, with increases of 36.8% and 50.8%, respectively. ZC showed its greatest increase from −0.6 to −1.0 MPa, with an increase of 57.9%, whereas ZH-W exhibited its greatest increase from −0.2 to −0.6 MPa, with an increase of 65.7%.

#### 2.2.4. Two-Way ANOVA of the Effects of Drought Stress on *A. polyrhizum* Germplasms

Two-way ANOVA showed that drought stress had highly significant effects on all 11 measured indicators (*p* < 0.01) (Table 3). Germplasm effects were also highly significant for all indicators (*p* < 0.01). The drought stress × germplasm interaction had highly significant effects on SOD, POD, CAT, SP, SS, MDA, AGB, BGB, and SH (*p* < 0.01), whereas the interaction effects on PRO and RL were not significant (*p* > 0.05).

### 2.3. Comprehensive Evaluation of Drought Resistance in Five A. polyrhizum Germplasms

#### 2.3.1. Correlation Analysis of Drought Resistance Coefficients Among Measured Indicators of *A. polyrhizum* Germplasms

**Figure 8:**
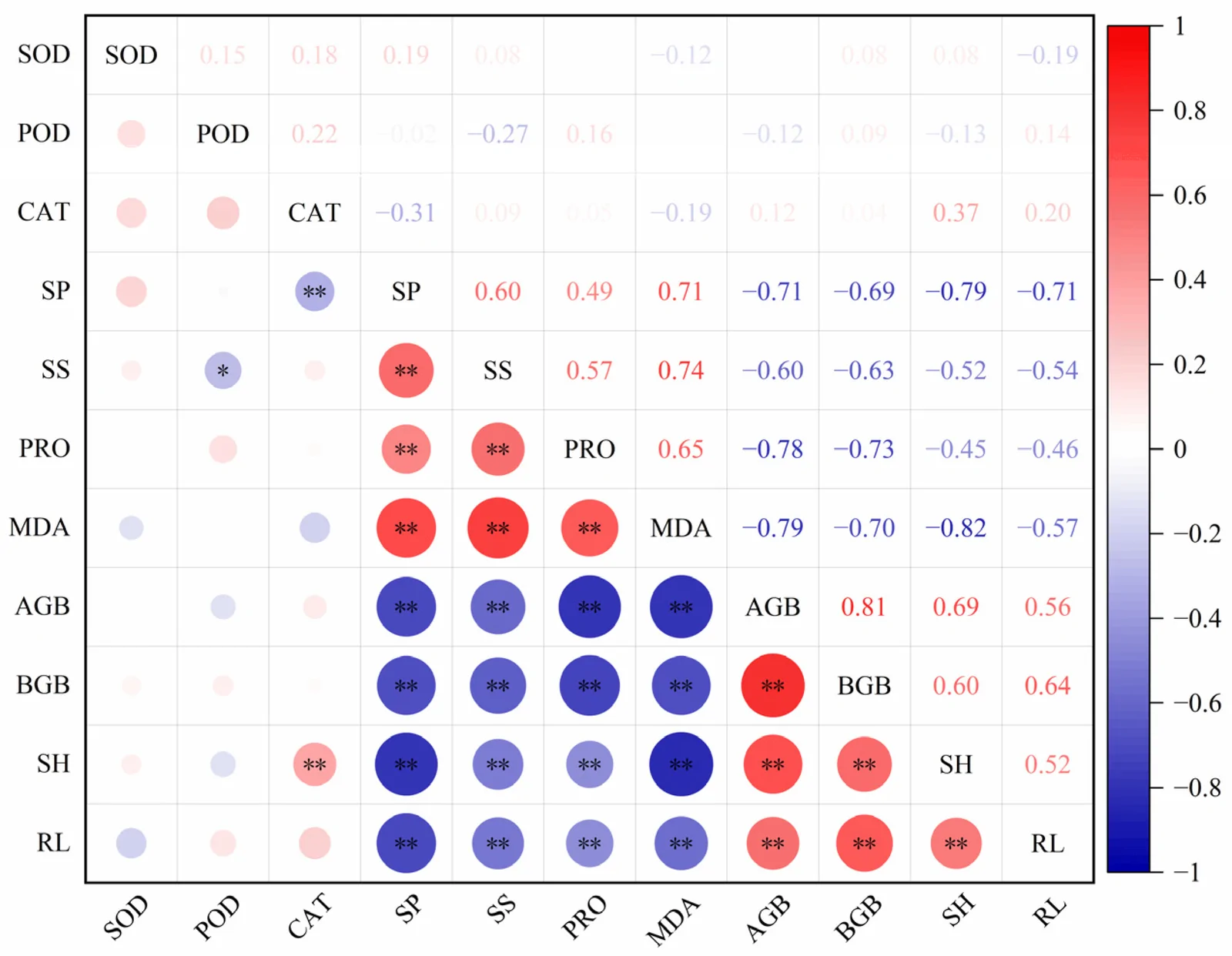
Correlation analysis among drought resistance coefficients of measured indicators of *A. polyrhizum* germplasms under drought stress. The size of the circles represents the absolute value of the correlation coefficient, with larger circles indicating stronger correlations. * *p* < 0.05; ** *p* < 0.01 (two-tailed). SOD, superoxide dismutase; POD, peroxidase; CAT, catalase; SP, soluble protein; SS, soluble sugar; PRO, free proline; MDA, malondialdehyde; AGB, aboveground biomass; BGB, belowground biomass; SH, shoot height; RL, root length.

Correlation analysis showed that the drought resistance coefficients of different indicators exhibited varying degrees of association (Figure 8). POD was negatively correlated with SS (*p* < 0.05), whereas CAT was negatively correlated with SP and positively correlated with SH (*p* < 0.01). SP, SS, PRO, and MDA were positively intercorrelated and negatively correlated with AGB, BGB, SH, and RL (*p* < 0.01). All phenotypic characteristics were significantly positively correlated with each other (*p* < 0.01), with the strongest correlation between AGB and BGB (*r* = 0.81).

#### 2.3.2. Principal Component Analysis of Drought Resistance Coefficients of Measured Indicators of *A. polyrhizum* Germplasms

Principal component analysis was performed on the drought resistance coefficients of the 11 measured indicators for the five *A. polyrhizum* germplasms under different drought stress treatments. Four principal components were retained under each treatment from −0.2 to −1.8 MPa, and the weight of PC1 was higher than those of the other principal components under all stress treatments, indicating that PC1 made the greatest contribution to the comprehensive evaluation of drought resistance under different stress conditions (Table 4 and Table 5). Under the −0.2 MPa treatment, the cumulative contribution rate of the four principal components was 80.45%, with relatively high loadings observed for MDA, RL, AGB, CAT, and SH. Under the −0.6 MPa treatment, the cumulative contribution rate was 81.98%, with relatively high loadings for SOD, SP, SS, MDA, and BGB. Under the −1.0 MPa treatment, the cumulative contribution rate was 83.88%, with relatively high loadings for SP, SH, and MDA. Under the −1.4 MPa treatment, the cumulative contribution rate was 86.46%, with relatively high loadings for POD, SP, PRO, CAT, SH, and SOD. Under the −1.8 MPa treatment, the cumulative contribution rate was 89.24%, with relatively high loadings for POD, SS, AGB, SP, PRO, MDA, and BGB.

#### 2.3.3. Comprehensive Drought Resistance Evaluation of Five *A. polyrhizum* Germplasms

The comprehensive evaluation based on the membership function showed considerable variation in D values among the five *A. polyrhizum* germplasms under different drought stress conditions (Table 6). Under the −0.2, −0.6, −1.0, −1.4, and −1.8 MPa treatments, the highest D values were observed for HC (0.74), ZH-W (0.88), ZH-P (0.85), ZH-W (0.76), and ZC (0.68), respectively. The mean D value across all stress treatments was used as the comprehensive drought resistance index for each germplasm. The mean D values ranged from 0.42 to 0.53, with ZH-W showing the highest value (0.53) and LQS the lowest (0.42). Accordingly, the drought resistance of the five germplasms was ranked as ZH-W > HC > ZH-P > ZC > LQS.

## 3. Discussion

### 3.1. Phenotypic Responses of A. polyrhizum Germplasms to Drought Stress

Plant growth and development are affected by drought stress, and responses to drought stress vary among species, genera, and cultivars [19]. The first reaction of plants to drought stress is a change in growth and morphology, including changes in biomass and leaf and root growth [20]. Yuan et al. [21] found that drought stress significantly inhibited seed germination and seedling growth of *A. polyrhizum*. In environments with limited water availability, the shoot biomass of alfalfa decreases [22]; oats respond to drought stress by reducing root growth [23]. In this study, after 40 days of drought stress, the shoot height, root length, and biomass of the five *A. polyrhizum* germplasms decreased to varying degrees as drought stress intensified (Table 1 and Table 2). Because of its harsh growing environment, *A. polyrhizum* bulbs are wrapped in thick, dead bulb scales to protect the bulbs and roots, forming a protective layer that prevents exposure to sunlight and reduces water evaporation [24]. Under drought stress, the growth of *A. polyrhizum* roots is inhibited, accompanied by corresponding reductions in shoot growth, which may help reduce the water demand associated with plant growth and enhance its survival under drought conditions. Different plant species exhibit distinct biomass allocation patterns under drought conditions, and shifts in biomass allocation can reflect their adaptive strategies [25].

### 3.2. Biochemical Responses of A. polyrhizum Germplasms to Drought Stress

**Figure 9:**
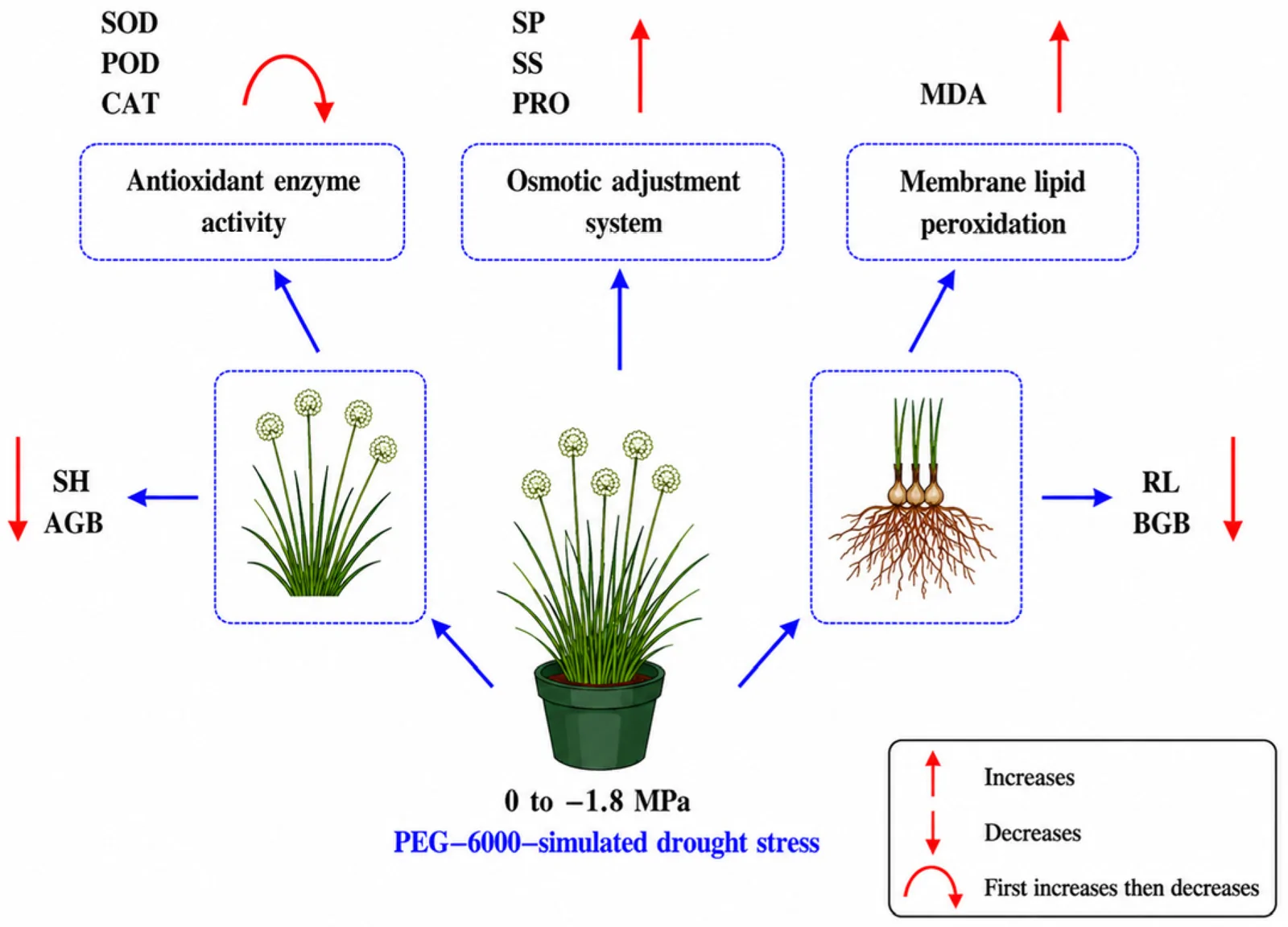
Phenotypic and biochemical responses of *A. polyrhizum* to PEG-6000-simulated drought stress. Blue arrows indicate the effects of drought stress on phenotypic and biochemical characteristics, whereas red arrows indicate the changing trends of the measured indicators. SOD, superoxide dismutase; POD, peroxidase; CAT, catalase; SP, soluble protein; SS, soluble sugar; PRO, free proline; MDA, malondialdehyde; AGB, aboveground biomass; BGB, belowground biomass; SH, shoot height; RL, root length.

During growth and development, water is one of the necessary conditions for plant growth, especially for drought-resistant plants. Changes in water availability not only affect plant phenotypic characteristics but also induce changes in biochemical characteristics [26]. *A. polyrhizum* responds to drought stress through various phenotypic adjustments and biochemical regulatory mechanisms. To illustrate these responses, we constructed a phenotypic and biochemical response diagram of *A. polyrhizum* under PEG-6000-simulated drought stress (Figure 9). Overall, *A. polyrhizum* responds to drought stress through changes in various phenotypic and biochemical processes, including growth adjustment, regulation of antioxidant enzyme activities, and osmotic regulation mechanisms.

Research has shown that the main antioxidant enzymes in plants are superoxide dismutase, peroxidase, and catalase, whose primary roles are to eliminate harmful reactive oxygen species in plants and protect the plant membrane system [27,28]. Our study results indicate that under drought stress conditions, as drought stress intensified, the activities of these three antioxidant enzymes generally increased initially and then decreased (Figure 1, Figure 2 and Figure 3). Under relatively low levels of drought stress, increased activities of superoxide dismutase, peroxidase, and catalase may help reduce damage to plant tissues, thereby effectively protecting the root system of *A. polyrhizum*. At these stress levels, *A. polyrhizum* may maintain its adaptive capacity through osmotic adjustment and regulation of the antioxidant defense system. However, as drought stress intensified, the activities of these antioxidant enzymes declined after reaching their respective peaks, suggesting that the protective capacity of the antioxidant defense system became limited under severe drought stress. Wang et al. [29] found that the antioxidant activity of potato plants under drought stress initially increased and then decreased. Sadeghi et al. [30] reported that drought stress affected the activities of antioxidant enzymes and proline content in different varieties of *Alcea rosea*, enabling them to adapt to semi-arid environments.

Soluble sugars, soluble proteins, and free proline are important osmotic adjustment substances in plants and play important roles in maintaining cellular osmotic balance, membrane stability, and plant adaptation to adverse environmental conditions [31,32]. This study found that the contents of soluble sugar, soluble protein, and free proline generally increased to varying degrees as drought stress intensified (Figure 4, Figure 5 and Figure 6). Malondialdehyde content is one of the important indicators reflecting the degree of damage to plants under adverse environmental conditions [33]. In this study, malondialdehyde content generally increased in the five *A. polyrhizum* germplasms as drought stress intensified (Figure 7), indicating a progressive increase in membrane lipid peroxidation. Particularly under severe drought stress at −1.8 MPa, malondialdehyde content increased markedly, whereas the activities of antioxidant enzymes, including superoxide dismutase, peroxidase, and catalase, declined after reaching their respective peaks. This may indicate that under severe drought stress, the capacity of the antioxidant defense system to scavenge reactive oxygen species became limited, resulting in excessive reactive oxygen species accumulation and enhanced membrane lipid peroxidation, thereby aggravating cell membrane damage. The results of this experiment were similar to those reported by Huang et al. [34] and Qin et al. [35] regarding the responses of *Allium* species to drought stress.

### 3.3. Effects of Drought Stress, Germplasm, and Their Interaction on A. polyrhizum

In this study, two-way ANOVA showed that drought stress and germplasm had highly significant effects on all 11 measured indicators (Table 3), indicating that the phenotypic and biochemical responses of *A. polyrhizum* were influenced not only by stress intensity but also by differences among germplasms. In addition, the interaction between drought stress and germplasm was highly significant for most indicators, further indicating that different germplasms responded differently to varying levels of drought stress. Gedam et al. [36] found that drought stress treatment and genotype significantly affected the phenotypic, physiological, and biochemical characteristics of onion (*Allium cepa*), while the genotype × environment interaction also had significant effects on multiple traits. Similarly, Shirvani et al. [37] reported that drought stress level, genotype, and their interaction significantly influenced phenotypic as well as physiological and biochemical indicators in wild barley. These findings are consistent with the results of the present study, indicating that plant responses to drought stress are strongly dependent on germplasm and that phenotypic and biochemical variation results from the combined effects of stress intensity and intrinsic differences among germplasms. Therefore, when evaluating the drought resistance of *A. polyrhizum* germplasms, the responses of multiple related indicators under different drought stress levels should be comprehensively considered to improve the reliability of drought resistance assessment.

### 3.4. Comprehensive Drought Resistance Evaluation of A. polyrhizum Germplasms

Correlation analysis of the drought resistance coefficients showed that the 11 indicators were correlated to varying degrees (Figure 8). Therefore, it is necessary to integrate principal component analysis and the membership function method for a comprehensive evaluation of multiple drought resistance indicators. The results of principal component analysis and membership function-based comprehensive evaluation indicated marked differences in drought resistance among the five *A. polyrhizum* germplasms collected from different habitats (Table 4, Table 5 and Table 6). The overall drought resistance ranking was ZH-W > HC > ZH-P > ZC > LQS, with ZH-W and HC exhibiting relatively strong drought resistance, whereas LQS showed comparatively weak drought resistance. The differences in drought resistance among germplasms may be related to long-term adaptation to their native habitats. ZH-W and ZH-P were both collected from rocky mountainous areas with an annual precipitation of 349.9 mm and shared the same community type. The relatively limited soil water-holding capacity in rocky mountainous habitats may impose long-term water-deficit selection pressure on plants, potentially favoring the development of stronger drought adaptation. In this study, ZH-W and ZH-P ranked first and third, respectively, in comprehensive drought resistance. However, their drought resistance still differed to some extent, suggesting that, in addition to habitat conditions, intrinsic differences among germplasms may also play an important role in drought resistance. HC originated from a loess hilly area with an annual precipitation of only 281 mm, the lowest among the five collection sites, and its native community type was *Reaumuria soongorica* + *Ajania* sp. *Reaumuria soongorica* is a common xerophytic species in arid and semi-arid regions [38]. Long-term growth under low-precipitation and relatively arid conditions may have contributed to the strong drought adaptation of HC. In contrast, ZC and LQS also originated from loess hilly areas, with annual precipitation of 325 and 293 mm, respectively, but ranked only fourth and fifth in comprehensive drought resistance. These results indicate that annual precipitation in the native habitat alone cannot determine the drought resistance of *A. polyrhizum* germplasms. Rather, drought resistance may result from the long-term combined effects of precipitation, topographic habitat conditions, and intrinsic differences among germplasms. Wang et al. [39] studied drought resistance during seed germination in these five *A. polyrhizum* germplasms and found that at an osmotic potential of −1.8 MPa, seeds of all five germplasms were able to germinate, with ZH-W seeds exhibiting relatively strong drought resistance, similar to the results of this study.

## 4. Materials and Methods

### 4.1. Experimental Materials

Five germplasms of *A. polyrhizum* (Table 7) were used as experimental materials, all sourced from Lanzhou City, Gansu Province, China. The collection area has a temperate continental climate with large temperature differences, low precipitation, and relatively mild climatic conditions. The annual average precipitation is 327 mm, and the annual average temperature is 10.3 °C. In early August 2023, *A. polyrhizum* germplasms from different habitats and community types were collected. The plants were placed in plastic bags, labeled, and transported to the experimental site. Plants of uniform size were selected and transplanted into plastic pots filled with cleaned sand. Each germplasm consisted of 18 pots, with four plants per pot, for a total of 90 pots (top diameter: 19 cm, height: 17.3 cm, bottom diameter: 14.2 cm). The experiment was conducted at Gansu Agricultural University in Yingmen Village, Anning District, Lanzhou, Gansu Province, China. The plants were pre-cultivated for one month, during which each pot received 250 mL of Hoagland nutrient solution prepared with distilled water every two days. Excess solution naturally flowed into the tray below, and other maintenance practices were consistent to ensure normal root growth of the plants. The plants were placed in a shed covered with plastic film to provide good ventilation and suitable temperature conditions.

### 4.2. Experimental Design

The pre-cultivated plants of each *A. polyrhizum* germplasm were randomly assigned to six treatment groups, with three replicates per treatment, for PEG-6000-simulated drought stress. Six drought stress levels were established: CK (normal watering) and PEG-6000 solutions with osmotic potentials of −0.2, −0.6, −1.0, −1.4, and −1.8 MPa. The control group received 250 mL of distilled water, whereas the treatment groups received 250 mL of PEG-6000 solutions at different concentrations. The PEG-6000 solutions were prepared according to Equation (1) [40]. The experiment lasted for 40 days. At the end of the 40-day treatment period, the *A. polyrhizum* plants were carefully removed from the plastic pots and quickly transported to the laboratory. The plants were then cleaned, blotted dry, and separated into aboveground and belowground parts, wrapped in aluminum foil, frozen in liquid nitrogen, and stored at −80 °C until analysis.

*Ψ_s_* = −(1.18 × 10^−2^) *C* − (1.18 × 10^−4^) *C*^2^ + (2.67 × 10^−4^) *CT* + (8.39 × 10^−7^) *C*^2^*T* (1)

where *Ψ_s_* is the osmotic potential of the solution (bar), 1 bar = 0.1 MPa; *C* is the concentration of PEG-6000; and *T* is the temperature (set at 25 °C for this experiment).

### 4.3. Determination of Indicators and Methods

Shoot height and root length were measured using a ruler. Biomass was determined using an analytical balance with a precision of 0.001 g. Biochemical indicators were measured in root tissues. Soluble sugar (SS) content was determined using the anthrone colorimetric method, soluble protein (SP) content using the Coomassie brilliant blue colorimetric method, free proline (PRO) content using the acid-ninhydrin method, and malondialdehyde (MDA) content using the thiobarbituric acid method [41]. Superoxide dismutase (SOD) activity was determined using the nitroblue tetrazolium method, peroxidase (POD) activity using the guaiacol method, catalase (CAT) activity using the hydrogen peroxide method [42]. Absorbance was measured using a SpectraMax^®^ iD3 Multi-Mode Microplate Reader (Molecular Devices, San Jose, CA, USA). Membership function values were calculated following the methods of Li et al. [43] and Xu et al. [44].

(2) $$Drought\;resistance\;coefficient=\frac{\left.measured\;value\;under\;drought\;treatment\right.}{measured\;value\;under\;CK\;treatment}$$

(3) $$R\left(V_{i}\right)=\frac{\left.V_{i}-V_{min}\right.}{\left.V_{max}-V_{min}\right.}$$

(4) $$W_{i}=\frac{P_{i}}{\sum_{i=1}^{m}P_{i}}$$

(5) $$D=\sum_{i=1}^{m}R\left(V_{i}\right)\times W_{i}$$

In these equations, $R\left(V_{i}\right)$ represents the membership function value of the *i*th comprehensive indicator; $V_{i}$ represents the score of the ith comprehensive indicator; $V_{min}$ and $V_{max}$ represent the minimum and maximum scores of the ith comprehensive indicator, respectively; $W_{i}$ represents the weight of the ith comprehensive indicator among all comprehensive indicators; $P_{i}$ represents the contribution rate of the ith comprehensive indicator obtained from principal component analysis; m represents the number of comprehensive indicators; and D represents the comprehensive drought resistance evaluation value.

### 4.4. Data Analysis

Data were organized using Microsoft Excel 2010 and are presented as means ± standard error (SE). Statistical analyses were performed using IBM SPSS Statistics 27.0, including one-way analysis of variance (one-way ANOVA) followed by Duncan’s multiple range test (*p* < 0.05), two-way analysis of variance (two-way ANOVA), Pearson correlation analysis, and principal component analysis (PCA). Principal components with eigenvalues greater than 1 were retained. Figures were generated using Origin 2021.

## 5. Conclusions

Drought stress inhibited shoot height and root length to varying degrees in the five *A. polyrhizum* germplasms collected from Longquansi Town (LQS), Hongcheng Town (HC), Zhongchuan Town (ZC), and Zhonghe Town (ZH-P and ZH-W) in Lanzhou, Gansu Province. In response to drought stress, *A. polyrhizum* modulated antioxidant enzyme activities and osmotic adjustment, thereby enhancing its capacity to cope with drought stress. Among the five *A. polyrhizum* germplasms, ZH-W exhibited the strongest drought resistance, followed by HC. We believe that *A. polyrhizum* can be used for ecological improvement and restoration of degraded grasslands in temperate arid and semi-arid regions.

## Figures and Tables

**Table 1:** Effects of drought stress on shoot height and root length of *A. polyrhizum* germplasms.

	Treatment (MPa)	LQS	HC	ZC	ZH-P	ZH-W
Shoot height (cm)	CK	15.30 ± 0.52 a	14.80 ± 0.21 a	17.80 ± 0.70 a	25.20 ± 3.37 a	23.10 ± 1.55 a
	−0.2	14.30 ± 0.26 a	13.47 ± 0.23 b	15.83 ± 0.32 b	19.20 ± 0.12 b	19.20 ± 1.14 b
	−0.6	12.47 ± 0.61 b	13.20 ± 0.42 b	13.67 ± 0.27 c	18.03 ± 0.39 b	15.20 ± 0.80 c
	−1.0	11.20 ± 0.40 b	12.10 ± 0.52 c	13.20 ± 0.50 c	16.47 ± 0.47 bc	12.13 ± 0.47 d
	−1.4	11.20 ± 0.21 b	12.17 ± 0.32 c	10.73 ± 0.29 d	15.33 ± 0.37 bc	11.63 ± 0.38 d
	−1.8	8.70 ± 0.40 c	10.93 ± 0.09 d	6.60 ± 0.91 e	12.50 ± 1.28 c	9.57 ± 0.70 d
Root length (cm)	CK	13.40 ± 1.01 a	13.80 ± 2.55 a	9.60 ± 0.95 a	12.90 ± 1.21 a	12.07 ± 0.41 a
	−0.2	11.33 ± 0.30 ab	12.40 ± 1.76 a	9.73 ± 1.19 a	10.93 ± 0.49 ab	9.87 ± 0.48 ab
	−0.6	9.43 ± 0.15 bc	11.83 ± 0.87 a	8.27 ± 0.59 a	10.80 ± 0.60 b	8.40 ± 1.29 b
	−1.0	9.00 ± 0.60 bc	10.57 ± 0.43 a	7.83 ± 0.74 a	10.07 ± 0.32 bc	8.33 ± 0.09 b
	−1.4	7.57 ± 0.75 cd	9.87 ± 0.29 a	7.73 ± 0.35 a	8.53 ± 0.39 cd	7.57 ± 0.92 bc
	−1.8	6.13 ± 1.28 d	9.33 ± 0.57 a	7.63 ± 0.15 a	6.57 ± 0.43 d	5.63 ± 0.71 c

LQS, Longquansi; HC, Hongcheng; ZC, Zhongchuan; ZH-P and ZH-W, Zhonghe, represent the collection sites of the *A. polyrhizum* germplasms and were used to designate the different germplasms. Different lowercase letters within the same column indicate significant differences among treatments (*p* < 0.05), whereas the same letter indicates no significant difference.

**Table 2:** Effects of drought stress on aboveground biomass and belowground biomass of *A. polyrhizum* germplasms.

	Treatment (MPa)	LQS	HC	ZC	ZH-P	ZH-W
Aboveground biomass (g)	CK	1.48 ± 0.28 a	2.28 ± 0.13 a	3.77 ± 0.20 a	2.10 ± 0.09 a	1.91 ± 0.06 a
	−0.2	1.16 ± 0.05 ab	1.97 ± 0.06 b	3.08 ± 0.22 b	1.97 ± 0.03 a	1.22 ± 0.07 b
	−0.6	0.91 ± 0.05 bc	1.43 ± 0.05 c	2.55 ± 0.19 bc	1.42 ± 0.10 b	1.19 ± 0.04 b
	−1.0	0.68 ± 0.03 cd	1.27 ± 0.16 c	2.21 ± 0.08 cd	1.07 ± 0.12 c	1.09 ± 0.17 b
	−1.4	0.58 ± 0.03 cd	0.86 ± 0.06 d	1.67 ± 0.28 d	0.94 ± 0.05 c	0.72 ± 0.05 c
	−1.8	0.41 ± 0.04 d	0.57 ± 0.02 e	0.40 ± 0.05 e	0.63 ± 0.04 d	0.57 ± 0.07 c
Belowground biomass (g)	CK	4.16 ± 0.51 a	8.90 ± 0.94 a	5.80 ± 0.29 a	5.24 ± 0.56 a	3.26 ± 0.30 a
	−0.2	3.46 ± 0.17 a	6.32 ± 0.64 b	5.89 ± 0.79 a	4.38 ± 0.21 b	2.75 ± 0.07 ab
	−0.6	2.43 ± 0.23 b	5.25 ± 0.38 bc	4.86 ± 0.02 ab	3.58 ± 0.23 b	2.55 ± 0.20 b
	−1.0	2.28 ± 0.21 bc	4.49 ± 0.14 cd	4.19 ± 0.31 bc	2.30 ± 0.08 c	1.97 ± 0.21 c
	−1.4	1.49 ± 0.11 cd	3.22 ± 0.57 d	3.25 ± 0.13 c	2.13 ± 0.22 c	1.13 ± 0.03 d
	−1.8	1.31 ± 0.06 d	3.10 ± 0.25 d	1.80 ± 0.26 d	1.54 ± 0.07 c	1.30 ± 0.06 d

LQS, Longquansi; HC, Hongcheng; ZC, Zhongchuan; ZH-P and ZH-W, Zhonghe, represent the collection sites of the *A. polyrhizum* germplasms and were used to designate the different germplasms. Different lowercase letters within the same column indicate significant differences among treatments (*p* < 0.05), whereas the same letter indicates no significant difference.

**Table 3:** Two-way ANOVA of measured indicators of *A. polyrhizum* germplasms under drought stress.

Indicator	Treatment	Germplasm	Treatment × Germplasm
SOD	134.77 **	124.03 **	18.51 **
POD	422.59 **	66.50 **	11.26 **
CAT	27.67 **	6.26 **	5.06 **
SP	234.76 **	24.02 **	6.13 **
SS	164.43 **	82.86 **	5.80 **
PRO	159.45 **	38.45 **	1.52 ns
MDA	590.54 **	79.41 **	14.24 **
AGB	142.37 **	117.71 **	7.69 **
BGB	76.54 **	75.77 **	3.09 **
SH	75.92 **	43.12 **	3.95 **
RL	22.09 **	9.84 **	0.75 ns

Values are F-statistics. SOD, superoxide dismutase; POD, peroxidase; CAT, catalase; SP, soluble protein; SS, soluble sugar; PRO, free proline; MDA, malondialdehyde; AGB, aboveground biomass; BGB, belowground biomass; SH, shoot height; RL, root length. ** indicates significance at *p* < 0.01; ns indicates no significant effect (*p* ≥ 0.05).

**Table 4:** Principal component analysis of drought resistance coefficients of *A. polyrhizum* germplasms under drought stress.

Treatment (MPa)	PC	EV	CR (%)	CCR (%)	*W* *_i_*
−0.2	1	3.08	28.00	28.00	0.35
	2	2.66	24.15	52.15	0.30
	3	2.08	18.92	71.07	0.24
	4	1.03	9.38	80.45	0.12
−0.6	1	4.36	39.66	39.66	0.48
	2	2.31	20.99	60.66	0.26
	3	1.21	11.00	71.65	0.13
	4	1.14	10.33	81.98	0.13
−1.0	1	3.48	31.66	31.66	0.38
	2	2.55	23.21	54.87	0.28
	3	1.93	17.50	72.37	0.21
	4	1.27	11.52	83.88	0.14
−1.4	1	3.53	32.12	32.12	0.37
	2	3.12	28.37	60.49	0.33
	3	1.53	13.95	74.43	0.16
	4	1.32	12.03	86.46	0.14
−1.8	1	3.52	32.04	32.04	0.36
	2	3.08	27.98	60.02	0.31
	3	1.98	18.03	78.06	0.20
	4	1.23	11.19	89.24	0.13

PC, principal component; EV, eigenvalue; CR, contribution rate; CCR, cumulative contribution rate; W*_i_*, weight of the *i*th principal component.

**Table 5:** Principal component loading matrix of drought resistance coefficients of *A. polyrhizum* germplasms under drought stress.

Treatment (MPa)	PC	SOD	POD	CAT	SP	SS	PRO	MDA	AGB	BGB	SH	RL
−0.2	1	−0.644	0.517	0.280	−0.689	−0.264	0.305	0.736	0.366	0.615	0.116	0.785
	2	−0.573	−0.636	0.254	−0.559	0.393	0.465	−0.216	0.737	−0.584	−0.391	−0.306
	3	0.196	−0.127	0.702	0.117	−0.457	0.652	−0.471	−0.063	−0.164	0.777	0.174
	4	0.234	−0.290	0.384	−0.016	0.557	0.153	0.054	−0.396	0.251	−0.274	0.338
−0.6	1	0.945	−0.309	−0.557	0.861	0.743	0.379	−0.887	−0.324	0.003	−0.613	−0.600
	2	−0.014	0.615	−0.631	0.093	−0.307	−0.632	0.161	0.262	0.741	−0.599	−0.163
	3	−0.043	0.265	0.474	−0.171	0.460	0.190	−0.356	0.124	0.580	−0.116	0.380
	4	0.156	0.580	0.043	−0.248	−0.260	0.413	−0.042	−0.484	0.106	0.227	−0.418
−1.0	1	0.412	−0.662	−0.605	0.858	0.400	−0.614	−0.081	−0.430	−0.469	−0.805	−0.418
	2	−0.563	−0.639	−0.371	−0.390	−0.235	−0.164	0.720	−0.362	−0.693	0.445	0.359
	3	−0.617	−0.060	−0.014	−0.137	0.487	−0.614	−0.588	0.337	0.053	−0.178	0.644
	4	−0.002	−0.303	0.573	0.110	0.630	0.316	0.155	0.347	−0.399	0.114	−0.146
−1.4	1	0.087	−0.765	−0.125	0.821	0.599	−0.779	−0.452	−0.310	−0.659	−0.544	−0.504
	2	0.495	−0.285	0.855	−0.130	0.488	0.387	0.655	−0.396	−0.601	0.779	−0.324
	3	0.730	0.489	−0.301	0.421	−0.210	0.379	−0.039	−0.417	0.077	−0.188	−0.300
	4	0.350	0.115	0.357	−0.166	0.486	0.080	−0.557	−0.145	0.112	−0.199	0.638
−1.8	1	−0.663	0.910	−0.120	−0.107	−0.735	0.073	−0.218	−0.825	−0.418	−0.628	0.624
	2	0.161	−0.096	0.532	−0.873	0.141	0.924	0.391	−0.383	0.058	0.645	0.638
	3	0.584	0.318	−0.506	0.298	−0.587	0.218	0.868	−0.038	0.091	0.136	−0.155
	4	0.218	0.175	0.508	0.337	0.035	0.028	−0.047	−0.234	0.792	−0.297	0.077

PC, principal component; SOD, superoxide dismutase; POD, peroxidase; CAT, catalase; SP, soluble protein; SS, soluble sugar; PRO, free proline; MDA, malondialdehyde; AGB, aboveground biomass; BGB, belowground biomass; SH, shoot height; RL, root length.

**Table 6:** D values for drought resistance evaluation of *A. polyrhizum* germplasms under drought stress.

Germplasm	−0.2 MPa	−0.6 MPa	−1.0 MPa	−1.4 MPa	−1.8 MPa	Mean D	Overall Rank
LQS	0.25	0.48	0.43	0.54	0.38	0.42	5
HC	0.74	0.28	0.55	0.61	0.42	0.52	2
ZC	0.43	0.50	0.28	0.26	0.68	0.43	4
ZH-P	0.57	0.41	0.85	0.30	0.14	0.45	3
ZH-W	0.17	0.88	0.64	0.76	0.22	0.53	1

D, comprehensive drought resistance value calculated using the membership function method. A higher D value indicates stronger comprehensive drought resistance.

**Table 7:** Germplasm resources of *A. polyrhizum*.

Code	Site	Longitude	Latitude	Elevation (m)	Original Habitat	Flower Color	Community Type	Precipitation (mm)
LQS	Longquansi Town	103°44′ E	36°43′ N	1893	Loess hills	Purple	*Allium polyrhizum*	293
HC	Hongcheng Town	103°53′ E	36°49′ N	1903	Loess hills	Purple	*Reaumuria soongorica* + *Ajania* sp.	281
ZC	Zhongchuan Town	103°60′ E	36°45′ N	1862	Loess hills	Purple	*Artemisia* sp. + *Allium polyrhizum*	325
ZH-P	Zhonghe Town	103°46′ E	36°13′ N	1696	Rocky mountains	Purple	*Leymus secalinus* + *Allium polyrhizum*	349.9
ZH-W	Zhonghe Town	103°46′ E	36°13′ N	1705	Rocky mountains	White	*Leymus secalinus* + *Allium polyrhizum*	349.9

## Data Availability

With regard to the request for raw data, we would like to clarify that although the data is not stored in the public repository for reasons, we have a strict data availability policy. The data sets used and analyzed in this study can be obtained from the corresponding authors on reasonable request.
